# Role of the Adrenal Medulla in Hypoglycaemia-Associated Autonomic Failure—A Diabetic Perspective

**DOI:** 10.3390/metabo14020100

**Published:** 2024-01-31

**Authors:** Manjula Senthilkumaran, Coen Koch, Mauritz Frederick Herselman, Larisa Bobrovskaya

**Affiliations:** Health and Biomedical Innovation, Clinical and Health Sciences, University of South Australia, Adelaide, SA 5000, Australia

**Keywords:** hypoglycaemia-associated autonomic failure, hypoglycaemia, diabetes, adrenal, catecholamines

## Abstract

Hypoglycaemia-associated autonomic failure (HAAF) is characterised by an impairment in adrenal medullary and neurogenic symptom responses following episodes of recurrent hypoglycaemia. Here, we review the status quo of research related to the regulatory mechanisms of the adrenal medulla in its response to single and recurrent hypoglycaemia in both diabetic and non-diabetic subjects with particular focus given to catecholamine synthesis, enzymatic activity, and the impact of adrenal medullary peptides. Short-term post-transcriptional modifications, particularly phosphorylation at specific residues of tyrosine hydroxylase (TH), play a key role in the regulation of catecholamine synthesis. While the effects of recurrent hypoglycaemia on catecholamine synthetic enzymes remain inconsistent, long-term changes in TH protein expression suggest species-specific responses. Adrenomedullary peptides such as neuropeptide Y (NPY), galanin, and proenkephalin exhibit altered gene and protein expression in response to hypoglycaemia, suggesting a potential role in the modulation of catecholamine secretion. Of note is NPY, since its antagonism has been shown to prevent reductions in TH protein expression. This review highlights the need for further investigation into the molecular mechanisms involved in the adrenal medullary response to hypoglycaemia. Despite advancements in our understanding of HAAF in non-diabetic rodents, a reliable diabetic rodent model of HAAF remains a challenge.

## 1. Introduction

Worldwide, 422 million adults, constituting 8.5% of the adult population, were reported to be living with diabetes in 2014 and it was the seventh leading cause of death in 2016 [1]. Glucose homeostasis is of major importance to normal physiological function. Treatment with insulin or oral hypoglycaemic agents helps to restore normal blood glucose level and prevent some of the complications of hyperglycaemia. However, while attempting to achieve an optimal blood glucose level, patients often experience hypoglycaemia due to multiple reasons including accidental overdose. Hypoglycaemia becomes a fact of life for diabetes patients and significantly reduces their quality of life [2,3,4]. Type 1 diabetes patients experience thousands of episodes of hypoglycaemia over their lifetime and on average at least one such episode per year is severe with seizure or coma [5]. In people with type 1 diabetes, 2–4% of deaths have been associated with hypoglycaemia [6]. Hypoglycaemia requiring emergency assistance from health service personnel is also common in people with type 2 diabetes treated with insulin [7], and thus has a significant economic impact on the health care system [8].

**Figure 1:**
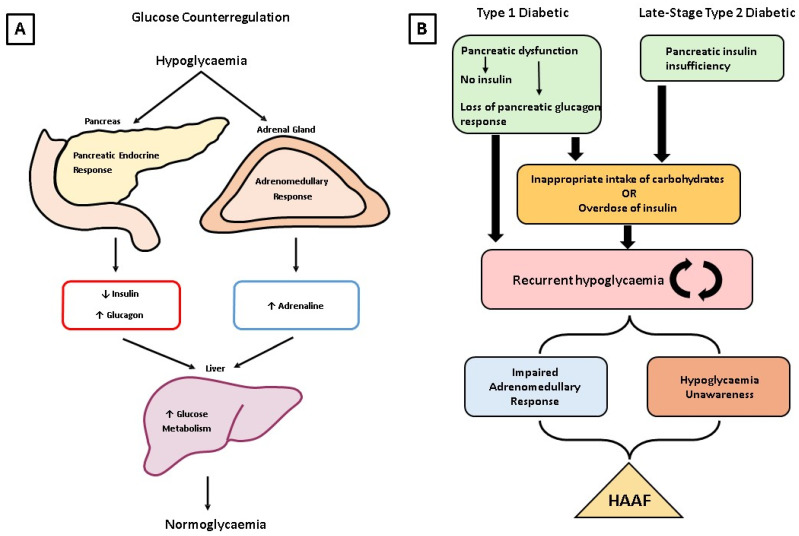
(**A**) The glucose counterregulatory response to hypoglycaemia in non-diabetic individuals. Following hypoglycaemia stimuli, the pancreatic endocrine response leads to reduced insulin and elevated glucagon, while the adrenal medulla releases adrenaline into the circulation. These molecules have a profound impact on glucose metabolism in the liver, which brings blood glucose levels back up to normal. (**B**) The progression of HAAF in a type 1 or type 2 diabetic patient. A type 1 diabetic will use exogenous insulin administration to treat the loss of pancreatic beta cells, while some type 2 diabetics may require exogenous insulin due to the progression of the disease. However, overdose of insulin can lead to hypoglycaemia. Concurrently, loss of pancreatic beta cells leads to alpha cell dysfunction and subsequent loss of the pancreatic glucagon response to hypoglycaemia in type 1 diabetics. In type 2 diabetics, insulin resistance results in a compensatory response by the pancreas to produce more insulin, leading to hypoglycaemia. Delayed postprandial insulin secretion can also occur in type 2 diabetics, increasing hypoglycaemic risk. In the longer term, type 2 diabetics rely on the administration of exogenous insulin due to the development of absolute insulin deficiency, placing them at risk of hypoglycaemic episodes with inappropriate overdoses of exogenous insulin. In both types of diabetes, this culminates in the onset of recurrent hypoglycaemia, which further impairs the adrenomedullary response and neurogenic symptoms response. This leads to hypoglycaemia-associated autonomic failure (HAAF), where more frequent and severe hypoglycaemic episodes can cause seizures, coma, and death.

In non-diabetic subjects, hypoglycaemia initiates an array of physiological responses called counterregulatory responses to establish normal blood glucose levels (please see reviews [9,10]; Figure 1A). Briefly, hypoglycaemia triggers a reduction in insulin secretion and an increase in glucagon secretion from the pancreas leading to increased glycogen breakdown and glucose production in the liver. A further drop in blood glucose initiates the sympathoadrenal response and neurogenic and neuroglycopenic symptom responses. The sympathoadrenal response results in the release of adrenaline from the chromaffin cells of the adrenal medulla. Adrenaline increases hepatic glucose production and reduces peripheral glucose utilisation. Neurogenic symptoms, such as hunger, sweating, and anxiety, stem from the activation of the sympathetic nervous system and ensure an individual is aware of a hypoglycaemic episode, prompting the individual to consume carbohydrate, termed hypoglycaemia awareness. Conversely, neuroglycopenic symptoms are a result of impaired brain function following neuroglycopenia and can include impaired cognitive function, and changes in mood and emotional state [11,12].

Type 1 diabetic patients will exhibit an absent endogenous insulin response, in combination with a glucagon response to hypoglycaemia that is lost within 5 years of the onset of diabetes [13,14]. Interestingly, the loss of glucagon response in type 1 diabetes seems to be limited to the hypogycaemia stimulus only, because other stimuli like insulin withdrawal, lipopolysaccharide exposure or exercise can still cause a significant glucagon response, although it was reduced when compared to non-diabetic individuals [15]. The same review also suggests that the loss of glucagon response to hypoglycaemia in type 1 diabetes may be irreversible. Type 2 diabetic patients do not develop a “true” glucagon deficiency; however, the response to glucagon may be altered following recurrent hypoglycaemia [6]. As such, diabetic patients rely on the secretion of adrenaline from the adrenal medulla, the sympathoadrenal response (discussed further below), and the onset of symptom responses to overcome hypoglycaemia. Patients with type 1 diabetes are more likely to experience hypoglycaemic episodes compared to type 2 diabetics, although equivalent rates are seen in type 2 diabetics on long-term insulin therapy [16,17]. However, recurrent hypoglycaemia resets the glycaemic threshold required to initiate the adrenomedullary and neurogenic symptom responses to a lower glucose level. In type 1 diabetics, this leads to the onset of severe neuroglycopenic symptoms such as seizure and coma prior to the onset of adrenomedullary and/or neurogenic symptom responses. In type 2 diabetes, this only occurs following the development of absolute insulin deficiency as a result of a gradual reduction in the secretion of insulin by the pancreas, thus these patients require exogenous insulin which controls plasma glucose levels, but does not alter pancreatic secretory processes of insulin and glucagon [16]. Thus, hypoglycaemic episodes become more frequent in type 2 diabetics on exogenous insulin therapy as they approach this stage of insulin insufficiency rather than earlier on in the disease process. Philip Cryer has identified the phenomenon by which recurrent hypoglycaemia impairs adrenomedullary responses and neurogenic symptom responses to subsequent hypoglycaemia as hypoglycaemia-associated autonomic failure (HAAF; Figure 1B) [12]. Due to the gradual decline in glucose counterregulation in type 2 diabetes, HAAF appears much later in type 2 diabetics than in type 1 diabetics. The mechanism of this progressive sympathoadrenal impairment to hypoglycaemia in diabetes in general is unclear even though many hypotheses have been proposed. Interestingly, it has been shown that type 2 diabetics in the early phase of the disease show exaggerated sympathoadrenal responses and symptomatic responses to hypoglycaemic episodes, likely due to them not yet being in a state of pancreatic insulin insufficiency [18]. Spontaneous hypoglycaemic events as a result of insulinomas are also known to disrupt counterregulatory responses and result in HAAF [19]. Currently, avoidance of hypoglycaemic episodes by relaxing intensive insulin treatment for several weeks is one of the approaches to restore sympathoadrenal responses, hypoglycaemia awareness or both [20,21]. Other therapies include patient education, novel diabetes technologies, pancreatic islet transplantation, and pharmaceutics. We will refer the readers to some excellent recent reviews covering these new therapies aiming to restore sympathoadrenal responses and/or hypoglycaemia awareness in diabetes [22,23].

However, attenuated hormonal response and hypoglycaemia unawareness are still a problem even with advancements in insulin delivery methods and novel diabetes technologies [23]. A recent study demonstrated that the use of a hybrid closed loop insulin delivery system was effective in improving symptom responses but failed to improve hormonal responses in type 1 diabetic patients. The hybrid closed loop insulin delivery system is an integrated device with a continuous blood glucose monitoring system, an insulin pump, and an algorithm that adjusts the dosage of insulin based on the data from the continuous glucose monitoring system [24]. Another study has shown that severe and moderate hypoglycaemia continue to occur in type 1 diabetes despite advanced diabetes technologies [25]. Also, patients using a continuous glucose monitoring system who develop hypoglycaemia unawareness still have a high risk to develop severe hypoglycaemia [26]. These findings thus emphasise the importance of more studies on the mechanism of HAAF phenomenon. Several excellent reviews are available on the possible mechanisms that impair the adrenomedullary responses and neurogenic symptom responses that comprise HAAF [27,28,29,30]. However, these reviews predominantly focus on the alterations at the level of central nervous system that can lead to HAAF. Despite a reduced adrenal medullary response being the hallmark characteristic of the HAAF phenomenon, studies investigating the role of the adrenal medulla in HAAF are minimal. Some of the previous studies (discussed below) have investigated whether a reduction in adrenal sympathetic nerve activity, overstimulation of acetylcholine receptors in the adrenal chromaffin cells, reduction in catecholamine synthesis, depletion of catecholamines or alterations in the mechanisms of adrenaline release from the adrenal gland play a role in diminished adrenaline response in HAAF [31,32,33,34,35,36,37]. Therefore, we will specifically review the work carried out so far to investigate the role of adrenal medulla in hypoglycaemia and alterations in the responses within the adrenal medulla in HAAF, the gaps in this knowledge, and what research is required to be undertaken in the future.

## 2. Pancreatic Responses to Hypoglycaemia and the Relevance of the Adrenal Gland

While the pancreas has a remarkable capability of counteracting hypoglycaemic states through stimulating the release of glucagon from pancreatic α-cells and decreasing insulin release from β-cells, its counterregulatory capacity is reliant on normal sensitivity to insulin. The importance of this is highlighted in patients with insulinomas which cause hyperinsulinemia since surgical removal of insulinomas has been shown to restore some parameters of the counterregulatory response to hypoglycaemia [38]. Yet, these patients do not show restoration of normal insulin sensitivity and even present with hypoglycaemic unawareness, a feature of HAAF [19,38,39].

Perturbations in insulin sensitivity in HAAF may be influenced by changes at the level of the adrenal gland. It is known that cortisol secreted by the adrenal glands acts on the pancreas to increase glucagon release and reduce insulin release [40]. Furthermore, it is known that the adrenal cortex and the adrenal medulla regulate the release of cortisol and adrenaline, respectively, in a synchronised bidirectional manner [41,42]. Given the fact that hypoglycaemic states increase the release of adrenaline from the adrenal medulla, this likely directly stimulates the release of cortisol from the adrenal cortex. However, recurrent hypoglycaemic states such as in HAAF diminishes the adrenaline response (as will be discussed later in this review), thus recurrent hypoglycaemia may diminish cortisol release, ultimately impacting the control of insulin release from the pancreas. Indeed, reduced cortisol levels have been reported in hyperinsulinemic hypoglycaemic patients, a frequent cause of recurrent hypoglycaemia [43]. These findings highlight the importance of the adrenal gland in the regulation of states of hypoglycaemia and suggest it is a key component to pathological states of recurrent hypoglycaemia.

## 3. Adrenal Medullary Responses to Hypoglycaemia

### 3.1. Overview of Adrenal Medulla Biology

The adrenal medulla is composed of modified post-ganglionic sympathetic neurons, called chromaffin cells, which are primarily responsible for the synthesis and release of catecholamines into the blood stream. Many similarities are shared between chromaffin cells and neurons, such as being derived from the neural crest during development and containing the necessary cellular machinery to synthesise and secrete catecholamines [44]. This has led to the extensive use of chromaffin cells in enhancing our understanding of neurophysiology [45]. Chromaffin cells are separated into two distinct phenotypes based on their catecholamine synthetic ability. In rats, adrenergic chromaffin cells which are capable of synthesising adrenaline can make up to 80% of the adrenal medulla, while 20% is made up of noradrenergic chromaffin cells, capable of synthesising noradrenaline but not adrenaline [46]; these two populations of the adrenal medulla can be selectively stimulated by different stressors [47]. The expression of the enzyme phenylethanolamine N-methyltransferase (PNMT), responsible for converting noradrenaline to adrenaline in the catecholamine synthetic pathway (will be elaborated further) determines a chromaffin cell’s phenotype. The unique ability to secrete adrenaline into the blood stream makes chromaffin cells the primary responders to stress within the body. Glucose deprivation is one such potential stressor that triggers the release of adrenaline from the adrenal medulla and a consequent increase in the plasma levels of adrenaline. The released adrenaline can function to increase plasma glucose during hypoglycaemia by promoting or inhibiting various biochemical processes across the body. The impact of adrenaline includes promotion of gluconeogenesis and glycogenolysis in the liver, promotion of glucagon secretion and reduction in insulin secretion in the pancreas, and reduced widespread glucose utilisation by peripheral cells [48]. A single episode of insulin-induced hypoglycaemia significantly increases plasma adrenaline levels in diabetic and non-diabetic rats and humans (see review [9]). However, the hallmark of the HAAF phenomenon is that prior exposure to hypoglycaemic episodes significantly diminishes this plasma adrenaline response to subsequent hypoglycaemia in non-diabetic and diabetic humans and rodents (see review [9]). Our focus in this review is on the adrenal medullary responses in HAAF. Below, we briefly describe general mechanisms of chromaffin cell activation, intracellular signalling in the chromaffin cells, and catecholamine synthesis. Additionally, we summarise what is known about the alterations within the adrenal gland in humans and rodents in response to single or recurrent hypoglycaemia and glucoprivation and highlight the gaps in our knowledge of HAAF.

### 3.2. Chromaffin Cell Activation, Signalling, and Catecholamine Release

#### 3.2.1. General Mechanisms

**Figure 2:**
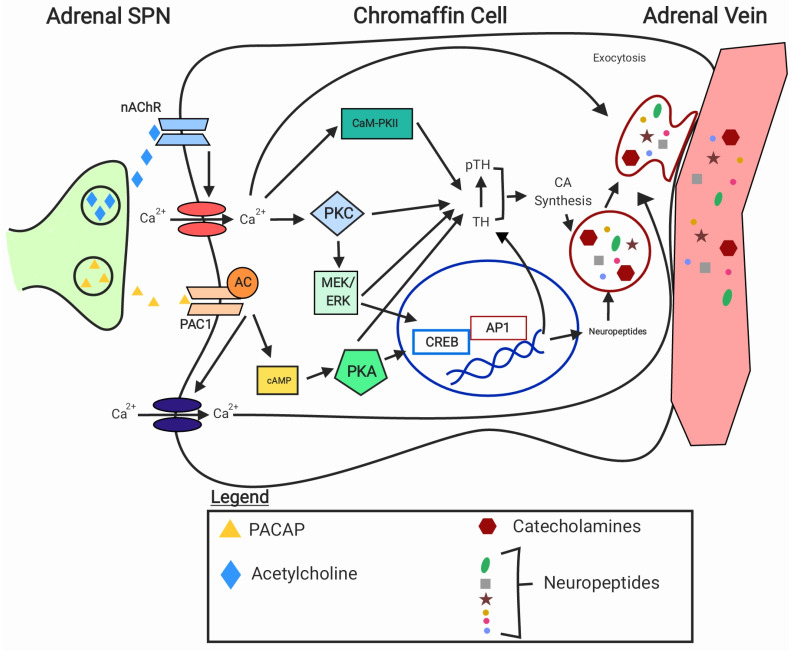
Chromaffin cell activation, signalling, and catecholamine release. Following activation of adrenal SPN by stimulus, acetylcholine and PACAP are released from the SPN nerve terminals in the synapse and bind to nAChR and PAC1 receptors, respectively, on the surface of chromaffin cells. Acetylcholine (Ach) is the primary neurotransmitter and causes cell depolarisation, opening of the voltage-gated calcium channels, and calcium entry followed by calcium-dependent exocytosis of catecholamines (CAs). The influx of calcium also stimulates various signaling pathways to increase tyrosine hydroxylase phosphorylation (pTH) and activation, and CA synthesis to replenish the released CAs. PACAP is another major neurotransmitter found in the adrenal SPNs but unlike Ach, it causes only a minor cellular depolarisation, and the resulting calcium entry of a low magnitude. Activation of PAC1 receptors, in association with adenyl cyclase (AC) by PACAP leads to cAMP-dependent activation of PKA, leading to the activation of CREB/AP1 and consequent induction of TH and neuropeptide mRNA levels. The newly synthesised neuropeptides are packaged into vesicles and co-released together with CAs in the bloodstream upon demand. Adrenal SPNs also contain a variety of other neuropeptides; however, their role in response to stress is still to be established. SPN, sympathetic preganglionic neurons; PACAP, pituitary adenylate cyclase-activating polypeptide; nAChR, nicotinic acetylcholine receptors; PAC1, PACAP type 1; cAMP, cyclic adenosine monophosphate; PKA, protein kinase A; PKC, protein kinase C; MEK/ERK, mitogen-activated protein kinase/extracellular signal-related kinase/; CREB, cyclic amp-response element binding protein; AP1, activator protein 1; CaM—PKII, Ca2+/calmodulin—protein dependent kinase II.

The adrenal medullas are linked to the central nervous system through specialised sympathetic preganglionic neurons (SPN; Figure 2). In rats, chromaffin cells are stimulated by specific SPN that arise predominately from the intermediolateral cell column within the T4–T12 thoracic spinal segments [49,50]. Upon activation, the SPN release neurotransmitters in the synapse. The main neurotransmitter causing catecholamine secretion from adrenal chromaffin cells is acetylcholine [51,52,53]. The nerve fibres innervating adrenal chromaffin cells express choline acetyltransferase, the enzyme involved in the synthesis of acetylcholine [54]. Binding of acetylcholine (ACh) to ACh nicotinic receptors on the chromaffin cells activates voltage-gated calcium channels, and the influx of calcium triggers the release of the secretory granules containing catecholamines and neuropeptides, through a process called exocytosis (see Figure 2). Pituitary adenylate cyclase activating peptide (PACAP) is another major neurotransmitter that is implicated in sustaining catecholamine secretion during stress [55,56]. PACAP contributes to catecholamine secretion via activation of PAC1 receptor, and the influx of calcium, although the resulting calcium entry is of low magnitude [57]. Studies investigating the neurochemical coding of the SPN reported that SPN innervating the adrenergic chromaffin cells express enkephalin and are activated by glucoprivation, whereas those innervating noradrenergic chromaffin cells express cocaine- and amphetamine-regulated transcript, and are sensitive to changes in blood pressure rather than glucoprivation [58,59]. Adrenal SPNs also contain a variety of other neuropeptides [60]; however, their role in response to stress is still to be established.

Binding of the neurotransmitters to the receptors on the chromaffin cells initiates signalling cascades involving several kinases (see Figure 2) including protein kinase A (PKA) and protein kinase C (PKC) and MAPKs [61,62,63]. These kinases are thought to phosphorylate some of the SNARE proteins (group of proteins that are necessary for membrane fusion and exocytosis of neurotransmitters), enabling them to form the SNARE complex, while the influx of calcium allows for activation of the complex, and thus the exocytosis of catecholamine-filled vesicles [64,65,66]. The SNARE protein complex consists of three essential proteins, synaptobrevin, syntaxin, and SNAP-25, and several regulatory proteins such as complexin and synaptotagmin [67]. Catecholamines, together with co-existing neuropeptides (see below for more information), are then released into the bloodstream for circulation. Breakdown of catecholamines within the chromaffin cells, liver, and kidney to their O-methylated metabolites normetanephrine and metanephrine occurs by the enzyme catechol-O-methyltransferase [68,69]. These metabolites are then converted by the enzyme monoamine oxidase to the waste product vanillymandelic acid, which is excreted in urine [70].

#### 3.2.2. Impact of Hypoglycaemia

Studies on possible alterations in the cell activation, signalling, and mechanisms of catecholamine release in the adrenal medulla after single and recurrent hypoglycaemia are limited and inconsistent, and can be summarised below.

##### 2-Deoxy Glucose and Insulin as Experimental Tools to Investigate the Effects of Hypoglycaemia and Glucoprivation

Experimentally, glucose deprivation can be caused by systemic administration of insulin or central/systemic administration of 2-deoxy glucose (2DG), an antiglycolytic glucose analogue. Within the cells, glucose is metabolised through glycolysis where it is initially converted to glucose-6-phosphate by hexokinase and metabolised further to produce energy in the form of ATP. 2DG is a derivative of glucose that is phosphorylated by hexokinase to 2-deoxy glucose-6-phosphate but does not metabolise further due to the missing 2-OH group. This inhibits glycolysis and glucose metabolism, thereby creating an energy deficit. Glucose is the primary fuel to the brain, and therefore a central energy deficit induced by 2DG triggers the glucose counterregulatory responses including endocrine and feeding responses in rats in a similar way as insulin-induced hypoglycaemia, thereby increasing blood glucose levels [61,71,72]. Since 2DG can cross the blood brain barrier, 2DG-induced neuroglycopenia can be achieved either through central or systemic administration. 2DG has thus been a very useful tool in investigating mechanisms involved in central glucose sensing [58,73,74,75,76,77,78]. However, there are several unique responses to hypoglycaemia that cannot be investigated using 2DG-induced neuroglycopenia [79]. A large number of studies have recently used insulin to induce single and recurrent hypoglycaemia as it is a more physiologically relevant stimulus to mimic the human HAAF phenomenon. However, the disadvantage of insulin-induced hypoglycaemia is that insulin can act directly on both the central nervous system and at the periphery, including the adrenal medulla, as insulin can cross the blood brain barrier and insulin receptors are expressed in the adrenal medulla [80]. Previous studies have shown that hyperinsulinemia increased adrenal sympathetic nerve activity in spontaneously hypertensive rats [81] and insulin dosage had a direct correlation to the magnitude of counterregulatory responses in humans [82]. Recurrent hyperinsulinemia itself significantly reduced glucose production and plasma adrenaline response to hypoglycaemia in normal rats [83]. In diabetic rats, the glucose production and plasma adrenaline response to hypoglycaemia were lower than the normal rats and recurrent hyperinsulinemia restored these responses to hypoglycaemia [84]. Regardless of the mode of glucose deprivation used, great difficulty has been seen in mimicking HAAF in not only healthy, but also type 1 diabetic patients [19]. Given their unique advantages and disadvantages, 2DG can be a great tool in investigating the mechanisms of glucose counterregulatory and feeding responses without the interference of insulin signalling, while insulin-induced hypoglycaemia can be used in elucidating the clinically relevant condition.

##### The Effects of 2DG and Insulin on Adrenaline Release in Humans and Rodents

In normal rats, systemic administration of 2DG significantly increased plasma adrenaline (within 20 min) as well as metanephrine (an adrenaline metabolite) levels [61,74]. Systemic administration of insulin increased plasma adrenaline levels in diabetic and nondiabetic rats and humans and this increase in plasma adrenaline is significantly blunted after recurrent hypoglycaemia (see reviews [9,85]). The plasma adrenaline level was not significantly reduced in response to subsequent insulin-induced hypoglycaemia in mice exposed to recurrent episodes of insulin-induced hypoglycaemia or 2DG induced-glucoprivation even though the urinary adrenaline level was significantly reduced in mice that had antecedent hypoglycaemia [34]. In diabetic rats not treated with insulin, the plasma adrenaline response to a single episode of insulin-induced hypoglycaemia was already significantly reduced compared to normal rats [84,86]. It is possible that chromaffin cell activation, intracellular signalling in the adrenal gland, catecholamine synthesis or the mechanism of catecholamine release is altered in diabetes. The plasma adrenaline level was further reduced in untreated diabetic rats exposed to 1–2 hypoglycaemic episode(s) per day for three consecutive days [84,87].

Nedoboy and Farnham [88] showed in their study that rats, injected with a single dose of streptozotocin to induce diabetes, did not show a reduction in the adrenaline counter regulatory response following recurrent (3 consecutive days) insulin injections in contrast to their non-diabetic counterparts. This result could potentially be explained by the fact that diabetic rats may have undergone some episodes of antecedent hypoglycaemia prior to the initiation of the HAAF protocol. The authors used insulin implants in diabetic rats that could potentially lead to undocumented antecedent hypoglycaemia. Interestingly, a counterregulatory glucagon response was not induced by single nor recurrent insulin injections in both diabetic rats and non-diabetic rats [88]. Thus, the HAAF model in diabetic rats requires further development and refinement to better reproduce the human condition.

In a validated mouse model of HAAF, C57Bl/6N mice were subjected to three protocols: saline injection, a single insulin injection, or five recurrent days of insulin injections [89]. The authors found that recurrent insulin injections lowered blood glucose and blunted glucagon and adrenaline responses [89]. To our knowledge this is the first study in mice that could successfully reproduce both components of the counterregulatory failure in response to recurrent hypoglycaemia. Thus, this study opens new opportunities for the research community to investigate the novel mechanisms of HAAF using genetically modified mice. Indeed, in the same study, the authors investigated the potential role of ghrelin in HAAF using ghrelin knockout mice and ghrelin cell-selective insulin receptor knockout mice and found that the counterregulatory responses (plasma glucagon and adrenaline) were reduced in both strains similar to the C57Bl/6N mice after recurrent hypoglycaemia. In a similar fashion, plasma ghrelin was also blunted in both the single and recurrent insulin injection groups [89]. The authors concluded that ghrelin deletion or elevation did not affect blood glucose or counterregulatory hormone responses, suggesting that ghrelin does not play a role in HAAF [89].

To investigate the impact that HAAF plays on adrenaline release in non-diabetic individuals, Lontchi-Yimagou et al. [90] experimentally induced HAAF using two separate protocols with varying success. The first, using a hyperinsulinemic hypoglycaemic clamp technique, found that, during antecedent hypoglycaemia, elevated plasma adrenaline was exhibited in healthy individuals susceptible to HAAF as opposed to those not susceptible. In the second protocol, the authors investigated a more novel approach to experimentally induce HAAF, through infusion of adrenaline in the absence of hypoglycaemia. This approach successfully replicated HAAF in non-diabetic individuals, while showing that plasma adrenaline levels dropped 27% in subsequent hypoglycaemic episodes. Similarly, a blunted counterregulatory and neurogenic symptom response has been demonstrated in healthy humans following three 2 h hypoglycaemic episodes over a 30 h duration with a reduction shown in adrenaline and cortisol levels [91].

In type 1 diabetic patients, the plasma adrenaline response to a single episode of hypoglycaemia was already reduced compared to normal subjects [92]. The authors state that this is likely due to reduced adrenaline stores and adrenaline secretory capacity of the adrenal gland as the level of plasma metanephrine in these patients was significantly reduced at baseline and during normoglycaemia compared to the healthy controls [92]. However, it is possible that the diminished baseline plasma adrenaline level is due to an undocumented hypoglycaemic episode(s) prior to the start of the experiment. Below, we summarise the studies investigating alterations in various aspects within the adrenal gland in response to single/recurrent hypoglycaemia.

##### The Effects of 2DG and Insulin on Adrenal Sympathetic Nerve Activity and Adrenomedullary Cell Activation

Systemic administration of both insulin and 2DG increased adrenal sympathetic nerve activity in rats [51,63]. Sivitz et al. [36] investigated if the reduced adrenaline response was due to reduced activity of the sympathetic nerves innervating the adrenal medulla. They exposed the rats to one episode of hypoglycaemia per day for two consecutive days and measured the activity of the nerves innervating the adrenal in response to a third episode on the next day by multifiber recording. The authors reported that the antecedent episodes of hypoglycaemia did not reduce the adrenal sympathetic nerve activity during subsequent episodes. In another study carried out by Herlein et al. [31], non-diabetic rats were subjected to two antecedent hypoglycaemic episodes and a subsequent transient hypotension induced by nitroprusside. The plasma catecholamine levels were determined before and after hypotensive episodes and the adrenal sympathetic nerve activity was continuously assessed by neural recording in the conscious state. The adrenal content of the catecholamines was also measured. These researchers report that antecedent hypoglycaemia blunted the subsequent adrenomedullary response to a non-hypoglycaemic stimulus and that this occurred in the absence of any impairment in sympathetic nerve traffic to the adrenal gland. However, since the adrenal gland catecholamine content was significantly reduced after antecedent hypoglycaemia, they presumed that this involved impaired catecholamine production and/or depleted adrenal catecholamine stores after antecedent hypoglycaemia [31]. In a different study, in rats exposed to 3–4 episodes of hypoglycaemia, the rate of catecholamine release in response to electrical stimulation of the splanchnic nerve was significantly reduced while the amount of catecholamine released in response to acetylcholine receptor agonists was not. Additionally, the catecholamine content of the adrenal gland as measured on the day after four episodes of hypoglycaemia was not significantly reduced. Therefore, this study concludes that the reduced adrenaline response to subsequent hypoglycaemia was more likely due to alterations in the signalling between the splanchnic nerve and the adrenal medulla [35]. One of the earlier studies showed that excessive stimulation of nicotinic acetylcholine receptors on the adrenal chromaffin cells during antecedent hypoglycaemia was the cause of diminished adrenaline response in HAAF since rats treated with cytisine, a nicotine receptor partial agonist, during antecedent hypoglycaemia showed significant improvement in the plasma adrenaline response [33]. Previous studies, including our own, had shown that Fos expression, a marker of cellular activity within the rat adrenal medullary cells increased significantly in response to 2DG-induced glucoprivation and insulin-induced hypoglycaemia [93,94]. Fos is the protein product of c-Fos, one of the immediate early proto-oncogenes. When cells are activated in response to a stimulus, an increase in Fos protein expression peaks at about two hours [95,96], which can be detected using immunohistochemical techniques. The Fos expression was reduced after ten episodes of glucoprivation in normal rats suggesting that the reduced adrenal medullary cell activation after recurrent glucoprivation may be contributing to the diminished plasma adrenaline response to subsequent glucoprivation [76]. Our group has previously demonstrated that activation of the adrenal medulla from insulin-induced hypoglycaemic stimulus occurs as high as adrenergic C1 and C3 neurons within the medulla in rats [94]. Further studies that utilised a rodent model of HAAF found that the activation of these C1 and C3 adrenergic neurons was impaired, demonstrated by a plasma adrenaline response that was significantly reduced in subsequent hypoglycaemic episodes [97].

##### The Effects of 2DG and Insulin on the Adrenomedullary Cell Signalling

In our own studies, glucoprivation increased the activation of some of the crucial signalling proteins including PKA, mitogen-activated protein kinase (MAPK), and cyclin-dependent kinase (CDK), and hypoglycaemia increased the activation of extracellular signal regulated protein kinase 1/2 (ERK 1/2) within the adrenal medulla [61,98]. The activation of ERK 1/2 was not affected after three episodes of hypoglycaemia in our laboratory [98]. In rats, the activation of PKA was not reduced after one or two hypoglycaemic episodes per day for three days [32], but recurrent hypoglycaemia has downregulated the gene expression of PKC in the adrenal medulla [37]. However, the effects of recurrent glucoprivation/hypoglycaemia on the other signalling proteins are yet to be investigated.

##### The Effects of 2DG and Insulin on Synaptic Proteins/Exocytosis

A recent study by Kim et al. [37] reported that genes of the proteins involved in synaptic contact and exocytosis such as SNAP-25 and synaptotagmin were downregulated in response to recurrent hypoglycaemia. Therefore, it is possible the diminished adrenaline response could also be due to defects in the mechanisms of release of catecholamines from the adrenal medulla. Defects in the mechanisms of catecholamine release are perhaps the least studied regarding the impact of recurrent hypoglycaemia and there is no data available so far on the effects of recurrent hypoglycaemia on adrenomedullary proteins involved in exocytosis.

### 3.3. Catecholamine Synthesis

#### 3.3.1. General Mechanisms

**Figure 3:**
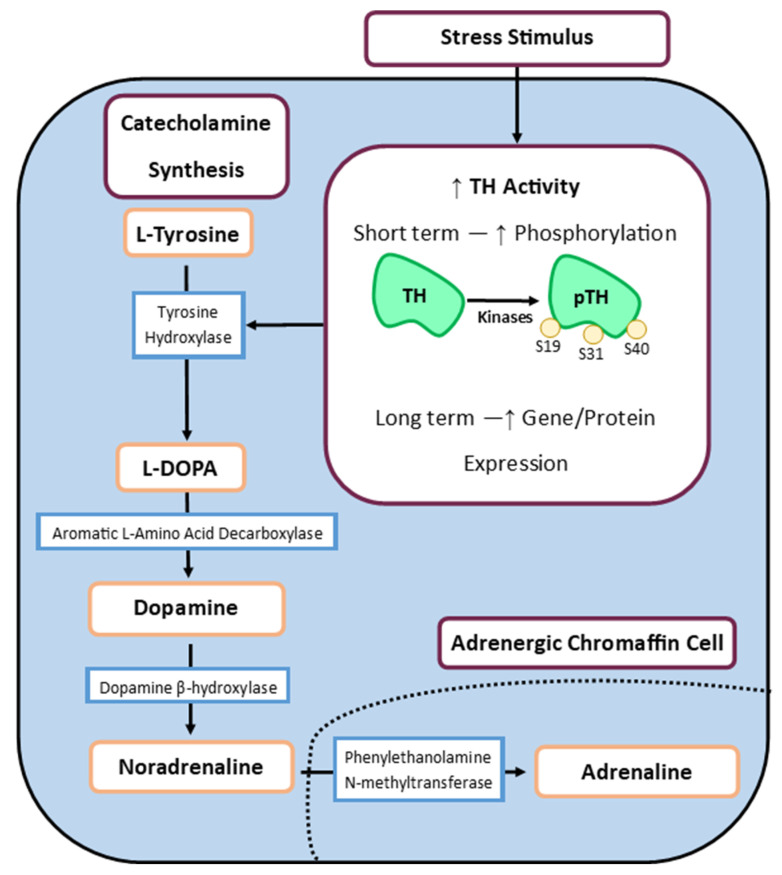
Regulation of catecholamine synthesis within chromaffin cells. Stimulation of the chromaffin cell by the splanchnic nerve leads to activation of both short-term and long-term mechanisms, which act to regulate catecholamine synthesis. In the short term, tyrosine hydroxylase (TH) is phosphorylated (pTH) by several kinases at three serine residues, Ser19 (S19), Ser31 (S31), and Ser40 (S40). Phosphorylation leads to TH activation (↑ TH activity). In the long term, both gene and protein expression are increased (↑ Gene/Protein expression). TH converts L-tyrosine to L-dihydroxyphenylalanine (L-DOPA). Aromatic L-amino acid decarboxylase (AADC) converts L-DOPA to dopamine. Dopamine β-hydroxylase (DBH) converts dopamine to noradrenaline. In adrenergic chromaffin cells only, noradrenaline is then converted to adrenaline by phenylethanolamine N-methyltransferase (PNMT).

After secretion, catecholamine stores are replenished through synthesis to ensure the chromaffin cell is ready for the next stress signal. This involves four key enzymes: tyrosine hydroxylase (TH), aromatic L-amino acid decarboxylase (AADC), dopamine β-hydroxylase (DBH), and PNMT, working in sequential fashion (Figure 3). Catecholamine biosynthesis begins with L-tyrosine uptake to chromaffin cells by specific L-type amino acid transporters, where the rate-limiting enzyme, TH, converts L-tyrosine to L-dihydroxyphenylalanine (L-DOPA). Decarboxylation of L-DOPA to dopamine by AADC follows, before being transferred inside temporary storage vesicles. Here, dopamine is converted to noradrenaline. The chromaffin cells that express PNMT can ultimately convert noradrenaline to adrenaline within the cytoplasm. Post-synthesis, catecholamines are transported into secretory granules by vesicular monoamine transporters (VMATs) and packaged with various neuropeptides, ready for exocytosis [99].

While all enzymes involved are highly regulated through short-term post-transcriptional modification or long-term protein expression, TH remains the most studied enzyme within the pathway. In the short term, TH activity is regulated by its phosphorylation at specific serine residues [61,100]. TH can be phosphorylated at serine residues 19, 31, and 40 [101]. Phosphorylation at Ser40 relieves feedback inhibition by catecholamines. Phosphorylation of TH at Ser40 causes a conformational change in TH that decreases the affinity of the bound catecholamines, and therefore increases their rate of dissociation. TH phosphorylation at Ser40 leads to a 40-fold increase in TH activity in vitro [100,102,103] and correlates with TH activity in vivo [104]. Phosphorylation at both Ser19 and Ser31 can also increase the rate of Ser40 phosphorylation, which can increase TH activity about two-fold [105,106,107]. In addition, evidence from recent studies indicates that Ser31TH phosphorylation can also increase TH activity in vivo [73,108]. In vitro, TH is phosphorylated by at least ten different protein kinases including cyclic AMP-dependent protein kinase, Ca^2+^/calmodulin—dependent kinase II (CaM–PKII), PKC, PKG, cell-cycle-dependent proline directed protein kinase, microtubule associated protein kinases, which are variously known as mitogen—activated protein kinases and extracellular signal regulated protein kinases and MAP kinase—activated protein kinases 1 and 2 [101]. In the long term, TH is regulated by increased TH protein synthesis [100].

#### 3.3.2. Impact of Hypoglycaemia in Diabetic and Non-Diabetic Rodents

The regulation of catecholamine synthetic enzymes is the most extensively studied aspect of the adrenal medulla; however, findings in relation to the impact of recurrent hypoglycaemia remain inconsistent. The catecholamine synthetic capacity of the adrenal medulla has previously been investigated by measuring TH phosphorylation in the short term, or gene and protein levels of TH in the long term, within the adrenal gland as described below. Some other studies have also investigated other catecholamine synthetic enzymes (see below).

##### Short-Term Changes—TH Phosphorylation

Many studies have investigated changes in TH phosphorylation, which increases TH activity and catecholamine synthesis. A study investigating TH phosphorylation in rats exposed to two episodes of insulin-induced hypoglycaemia per day for two days concluded that reduced catecholamine synthesis was the cause of reduced adrenaline response [32]. The authors of this study report that site-specific TH phosphorylation at Ser40 was significantly reduced in rats that had two hypoglycaemic episodes per day for 3 days prior to a subsequent hypoglycaemic clamp indicating reduced TH activity and catecholamine synthesis [32]. However, this study did not measure phosphorylation at Ser31, which could be an equally important mechanism to increase TH activity, and hence catecholamine synthesis in vivo [73,108]. Moreover, the reduction in Ser40TH phosphorylation was not consistent with the unaltered activation of PKA in their study [32]. In our own laboratory, one episode of 2DG-induced glucoprivation and insulin-induced hypoglycaemia increased site-specific TH phosphorylation at Ser31 and Ser40 within the adrenal medulla within one hour [61,109]. Moreover, in our studies, three episodes of insulin-induced hypoglycaemia did not reduce site-specific TH phosphorylation at Ser31 or Ser40 within the adrenal medulla when measured at 60 min after the last episode, suggesting that short-term regulation of catecholamine synthesis is unlikely to be affected in HAAF. This observation is consistent with ERK1/2 data in our study [98].

##### Mid-Term Changes—Gene Expression

Few studies have investigated catecholamine synthetic enzymes at the gene level (Table 1).

TH mRNA levels were found to increase within the adrenal gland of non-diabetic rats subjected to seven episodes of both repeated insulin-induced hypoglycaemia and 2DG-induced glucoprivation [79,110]. Whereas PNMT mRNA levels in response to subsequent hypoglycaemia was not affected in rats exposed to either one or two episodes of hypoglycaemia per day for 3 days [32]. Global gene expression profiling in the adrenal gland to identify variations in the expression of the genes after single and recurrent hypoglycaemia (two episodes per day for 3 days prior to subsequent hypoglycaemia) has highlighted that the expression of the gene that encodes PP2A, a common phosphatase that can dephosphorylate TH, is significantly increased after recurrent hypoglycaemia indicating faster deactivation of TH could occur after recurrent hypoglycaemia [37].

In untreated diabetic rats, the adrenal medullary TH mRNA level was significantly lower than the normal rats and PNMT mRNA levels were reduced in untreated diabetic rats exposed to two episodes of hypoglycaemia for 4 days (Table 1) [111].

##### Long-Term Changes—Total Protein Expression

Few studies have also investigated catecholamine synthetic enzymes at the protein level (Table 1). Our group had shown that TH protein expression within the adrenal medulla is increased 24 h following a single episode of 2DG-induced glucoprivation [61]. Additionally, we found that TH protein expression was increased in the adrenal medulla following three episodes of insulin-induced hypoglycaemia, when measured at 60 min after the last episode, suggesting that long-term regulation of catecholamine synthesis is unlikely to be affected in HAAF [98]. This is consistent with Kudrick et al.’s study showing increased TH protein in rats exposed to one hypoglycaemic episode per day for three days [32]. Furthermore, seven episodes of insulin-induced hypoglycaemia also increased TH protein levels within the adrenal gland in rats [110]. In contrast, Ma et al. [34] reported that TH immunoreactivity levels in the adrenal medulla in mice, rather than rats, as measured 24 h after a single episode of hypoglycaemia, was increased. However, after three episodes of hypoglycaemia, TH immunoreactivity was consistent with basal levels, reportedly due to the inhibitory effects of NPY on TH expression leading to reduced adrenaline response. This study also clarifies that the reduction in TH immunoreactivity was not due to altered cell proliferation or apoptosis of the chromaffin cells following hypoglycaemia. It is important to note that the significant reduction in plasma adrenaline which is the hallmark of the HAAF phenomenon was not achieved in Ma et al.’s study. In addition to TH, three episodes of insulin-induced hypoglycaemia also increased AADC and PNMT protein expression [98,112], while both recurrent glucoprivation and hypoglycaemia increased DBH protein expression within the adrenal gland [112]. All the above-mentioned results suggest that catecholamine synthesis is unlikely to be affected by recurrent hypoglycaemia.

**Table 1:** Alterations in the adrenal gland in response to recurrent glucoprivation/hypoglycaemia in diabetic and non-diabetic rodents. 2DG, 2-deoxy glucose, ASNA, adrenal sympathetic nerve activity; Ad, adrenaline; NAd, noradrenaline; AChR, acetylcholine receptor; ERK, extracellular signal regulated protein kinase; PKA, protein kinase A; PKC, protein kinase C; TH, tyrosine hydroxylase; DBH, dopamine β-hydroxylase; RH, single daily recurrent hypoglycaemia; 2RH, twice daily episodes of insulin-induced hypoglycaemia; PNMT, phenylethanolamine N-methyltransferase.

Alterations in the Adrenal Gland	Species	Recurrent 2DG/Insulin, Dose, and Route of Administration	Number of Antecedent Episodes	Finding in Response to Subsequent Glucoprivation/Hypoglycaemia	Time of Measurement	Plasma Adrenaline in Response to Single/Recurrent Hypoglycaemia
Catecholamine release	Non-diabetic mice	Insulin, 2.5 U/kg; i.p. [89]	Once a day for 5 days [89]	↓ adrenaline response [89]	30 min from the last injection [89]	~16/7 ng/mL [89]
	Non-diabetic rats	Insulin, 2 U/kg; i.p. [37]	Twice a day for 3 days [37]	↓ expression of genes of proteins associated with exocytosis [37]	60 min from the last injection [37]	Did not measure [37]
		Insulin, 5 U/kg; i.p. [88]	Once a day for 3 days [88]	↓ adrenaline response [88]	2 h from the last injection [88]	1317/170.7 pg/mL [88]
	Diabetic rats	Insulin, 30–40 IU/kg; i.p. [87]	Once a day for 3 days [87]	↓ adrenaline response [87]	90 min from the last injection [87]	5000/2500 pg/mL [87]
		Insulin, 5 U/kg; i.p. [88]	Once a day for 3 days [88]	↔ adrenaline response [88]	2 h from the last injection [88]	2958/3804 pg/mL [88]
Adrenal sympathetic nerve activity/ stimulation	Non-diabetic rats	Insulin, 1.5 U/kg; s.c. [36]	Once a day for 2 days [36]	↔ASNA [36]	During the subsequent hypoglycaemic episode [36]	~2800/1200 pg/mL [36]
		Insulin, 2 U/kg; s.c. [31]	Once a day for 2 days [31]	↔ASNA [31]	During the subsequent hypotensive episode [31]	~1100/700 pg/mL [31]
		Insulin, 1 U/kg; s.c. [35]	Once a day for 3 days [35]	a. ↓ Ad and NAd release in response to nerve stimulation; b. ↔ Ad and NAd release in response to AChR agonists [35]	a. Day 4; b. Day 5 [35]	707 ± 89/458 ± 135 pg/mL [35]
Chromaffin cell activation	Non-diabetic rats	2DG, 200 mg/kg; s.c. [76]	Once a day for 10 days [76]	Reduced Fos expression [76]	2 h from the last injection [76]	Did not measure [76]
Intracellular signalling	Non-diabetic rats	Insulin, 10 U/kg; i.p. [98]	Once a day for 2 days [98]	↔ ERK 1/2 activation [98]	60 min from the last injection [98]	4417 ± 594/2617 ± 185 pg/mL [98]
		Insulin, 2 U/kg; i.p. [32]	Once a day (RH) and twice a day (2RH) for 3 days [32]	↔ PKA activation [32]	60 min from the last injection [32]	~2/2 ng/mL (RH) ~2.8/1.5 ng/mL (2RH) [32]
		Insulin, 2 U/kg; i.p. [37]	Twice a day for 3 days [37]	↓ PKC gene expression [37]	60 min from the last injection [37]	Did not measure [37]
Adrenal gland catecholamine content	Non-diabetic rats	Insulin, 2 U/kg; s.c. [31]	Once a day for 2 days [31]	↓ Adrenaline content of the adrenal gland [31]	Measured on the day after the second hypoglycaemic episode [31]	~1100/700 pg/mL [31]
		Insulin, 1 U/kg; s.c. [35]	Once a day for 3 days [35]	↔ Adrenaline content of the adrenal gland [35]	Measured on the day after the subsequent hypoglycaemic episode [35]	707 ± 89/458 ± 135 pg/mL [35]
TH mRNA, protein, and phosphorylation	Non-diabetic rats [31,32,79,98,110,112]	2DG, 500 mg/kg and Insulin, 5 U/kg; i.p. [79]	Once a day for 6 days [79]	↑ TH mRNA [79] [110]	5 h after the seventh injection [79]	Did not measure [79]
		2DG, 500 mg/kg and Insulin, 5 U/kg; i.p. [110]	Once a day for 5 and 6 days [110]	↑ TH mRNA ↑ TH protein [110]	24 h after the sixth injection and 5 h after the seventh injection [110]	Did not measure [110]
		Insulin, 2 U/kg; i.p. [32]	Once a day (RH) and twice a day (2RH) for 3 days [32]	↓ TH mRNA (2RH) ↓ Ser40TH phosphorylation ↑ TH protein (2RH) [32]	TH mRNA: 3.5 h from the end of hypoglycaemic clamp on the last day; TH protein and pSer40TH at 60 min from the last injection [32]	~2/2 ng/mL (RH) ~2.8/1.5 ng/mL (2RH) [32]
		2DG, 500 mg/kg and Insulin, 2 U/kg; s.c. [112]	Once a day for 5 days [112]	↑ TH protein [112]	6 h after the administration of the last dose of 2DG/insulin [112]	Did not measure [112]
		Insulin, 10 U/kg; i.p. [98]	Once a day for 2 days [98]	↑ TH protein ↑ Ser40TH phosphorylation [98]	60 min from the subsequent episode [98]	4417 ± 594/2617 ± 185 pg/mL [98]
		Insulin, 2 U/kg; s.c. [31]	Once a day for 2 days [31]	↔ TH protein ↔ Ser40TH phosphorylation [31]	Day after the second hypoglycaemic episode [31]	~1100/700 pg/mL [31]
	Non-diabetic mice [34]	Insulin, 2.5 U/kg; i.p. [34]	Once a day for 3 days [34]	↓ TH immunoreactivity [20,34]	24 h after the last injection [34]	~7/4 ng/mL [34]
	Untreated diabetic rats [111]	Insulin, 2 U/kg; s.c. [111]	Twice a day for 4 days [111]	↔ TH mRNA [111]	Not given [111]	Did not measure [111]
	Insulin-treated diabetic rats [113]	Insulin, 2 U/kg; s.c. [113]	Twice a day for 3 days and one episode on day 4 [113]	↔ TH mRNA ↑ TH protein [113]	At 90 min during the subsequent episode [113]	~30/25 nM [113]
DBH Protein expression	Non-diabetic rats [98,112]	Insulin, 10 U/kg; i.p. [98]	Once a day for 2 days [98]	↔ DBH protein [98]	60 min from the subsequent episode [98]	4417 ± 594/2617 ± 185 pg/mL [98]
		2DG, 500 mg/kg and Insulin, 2 U/kg; s.c. [112]	Once a day for 5 days [112]	↑ DBH protein [112]	6 h after the administration of the last dose of 2DG/insulin [112]	Did not measure [112]
	Untreated diabetic rats [111]	2DG, 500 mg/kg and Insulin, 2 U/kg; s.c. [111]	Twice a day for 4 days [111]	↑ DBH mRNA [111]	Not given [111]	Did not measure [111]
	Insulin-treated diabetic rats [113]	2DG, 500 mg/kg and Insulin, 2 U/kg; s.c. [113]	Twice a day for 3 days and one episode on day 4 [113]	↓ DBH protein [113]	At 90 min during the subsequent episode [113]	~30/25 nM [113]
PNMT mRNA and protein expression	Non-diabetic rats [31,32,98,112]	Insulin, 2 U/kg; s.c. [31]	Once a day for 2 days [31,98]	↔ PNMT protein [31]	Day after the second hypoglycaemic episode [31]	~1100/700 pg/mL [31]
		Insulin, 2 U/kg; i.p. [32]	Once a day (RH) and twice a day (2RH) for 3 days [32]	↔ PNMT mRNA [32]	3.5 h from the end of hypoglycaemic clamp on the last day [32]	~2/2 ng/mL (RH) ~2.8/1.5 ng/mL (2RH) [32]
		Insulin, 10 U/kg; i.p. [98]	Once a day for 2 days [98]	↔ PNMT protein [98]	60 min from the subsequent episode [98]	4417 ± 594/2617 ± 185 pg/mL [98]
		2DG, 500 mg/kg and Insulin, 2 U/kg; s.c. [112]	Once a day for 5 days [112]	↑ PNMT protein (insulin) ↔ PNMT protein (2DG) [112]	6 h after the administration of the last dose of 2DG/insulin [112]	Did not measure [112]
	Untreated diabetic rats [111]	2DG, 500 mg/kg and Insulin, 2 U/kg; s.c. [111]	Twice a day for 4 days [111]	↓ PNMT mRNA [111]	Not given [111]	Did not measure [111]
	Insulin-treated diabetic rats [113]	2DG, 500 mg/kg and Insulin, 2 U/kg; s.c. [113]	Twice a day for 3 days and one episode on day 4 [113]	↔ PNMT mRNA and PNMT protein [113]	At 90 min during the subsequent episode [113]	~30/25 nM [113]

↓ indicates a decrease; ↑ indicates an increase; ↔, indicates no change.

In contrast, in insulin-treated diabetic rats, recurrent insulin-induced hypoglycaemic episodes did not reduce the plasma adrenaline response to subsequent hypoglycaemia. In insulin-treated diabetic rats, adrenal medullary TH protein level was increased after seven episodes of hypoglycaemia despite no increase in TH mRNA levels. In response to subsequent hypoglycaemia following seven antecedent hypoglycaemic episodes, TH, DBH, and PNMT protein levels were not reduced indicating that repeated insulin treatment in these rats preserved adrenaline response after recurrent hypoglycaemia mainly through preventing reductions in catecholamine synthetic enzymes [113]. This observation indicates that, apart from recurrent hypoglycaemia, diabetes and insulin therapy also have a significant impact on the catecholamine synthetic enzymes, and thus the adrenaline response to hypoglycaemia.

## 4. Possible Contribution of Adrenomedullary Peptides to HAAF

### 4.1. Overview of the Adrenomedullary Peptides and Their Role in Modulating Catecholamine Release

The main neurotransmitters causing catecholamine secretion from adrenal chromaffin cells are acetylcholine and pituitary adenylate cyclase activating polypeptide (PACAP), which are released from the axons of SPN that innervate the adrenal medulla [51,114]. In addition, a variety of neuropeptides such as vasoactive intestinal peptide (VIP), enkephalin (ENK), neuropeptide Y (NPY), galanin, substance P (SP), calcitonin gene-related peptide (CGRP), somatostatin and neurotensin coexist with catecholamines in adrenal chromaffin cells or occur in axons that innervate them as detailed below (Table 2).

Many of these peptides have been shown to regulate the secretion of catecholamines in vitro from the bovine and rat adrenal chromaffin cells but studies in humans are very limited. PACAP is found in rat and human adrenal medullary chromaffin cells [118,137,138] and stimulates catecholamine secretion from cultured chromaffin cells of many species including humans [56,117,118,119,120,121,138]. In rats, PACAP is expressed in both adrenaline-secreting and noradrenaline-secreting chromaffin cells [115] and in nerve fibres innervating them [139,140]. VIP is localised in both adrenaline- and noradrenaline-secreting chromaffin cells, intra-adrenal ganglion cells and nerve fibres, and increases the release of catecholamines from rat and human adrenal glands [118,122]. Bovine adrenal chromaffin cells, intra-adrenal ganglion cells, and nerve fibres in the adrenal gland in rats also contain NPY [123,126]. However, the effects of NPY on catecholamine secretion from adrenal chromaffin cells vary between species. In bovine chromaffin cells, NPY inhibits catecholamine secretion, whereas in human chromaffin cells in culture exogenous, NPY stimulates catecholamine secretion in a dose-dependent manner [124,125]. Neurotensin that occurs in noradrenaline-producing chromaffin cells and galanin that occurs in both adrenaline- and noradrenaline-producing chromaffin cells increase catecholamine secretion from bovine chromaffin cells and rat adrenal glands, respectively [127,129]. In contrast, the opioid peptide, ENK, which occurs in adrenergic chromaffin cells and SPN axons in the rat, inhibits the secretion of catecholamines from rat adrenal glands [130]. Furthermore, SP and CGRP inhibit nicotine-mediated catecholamine release from rat chromaffin cells [136] and somatostatin inhibits catecholamine release in guinea pigs [133]. Below, we will discuss how the gene/protein expression of specific peptides are altered in the adrenal medulla in response to single and recurrent hypoglycaemia.

### 4.2. Effects of Single and Recurrent Hypoglycaemia on the Gene Expression and Abundance of the Adrenomedullary Peptides

There are very few studies investigating the effects of insulin-induced hypoglycaemia on the gene expression or the protein expression of the peptides in the adrenal gland (See Table 3). One of them, undertaking global gene profiling in the adrenal gland, reported a significant increase in the expression of several adrenal peptides including NPY, galanin, proenkephalin, and PP2A, a common phosphatase that can dephosphorylate TH and AADC [101,141] in response to recurrent insulin-induced hypoglycaemia [37].

NPY gene expression within the adrenal medulla increased in a dose-dependent manner in response to insulin administration in rats. NPY mRNA levels in the adrenal medulla reached its peak within 8 h and returned to basal level in 48 h of insulin injection. NPY immunoreactivity increased within 30 min of insulin administration in the plasma and within 4 h in the adrenal medulla which remained elevated for several days [142,143,145]. In addition, NPY immunoreactivity within the adrenal gland in mice is increased in response to repeated hypoglycaemia [34]. Galanin mRNA levels were also significantly increased in the adrenal medulla in hypoglycaemic rats. A maximum increase was observed within 4 h of the insulin injection which returned to basal levels after 6 days of the treatment [146]. Galanin peptide content of the adrenal medulla, as measured by radioimmunoassay (RIA), increased more than 60-fold after 24 h in response to insulin-induced hypoglycaemia in rats [62]. Insulin treatment also increased adrenomedullary CGRP mRNA and peptide content within 24 h in rats as measured by RIA [142]. Another study in rats reported that neurotensin peptide content within the adrenal medulla increased by nearly 100-fold after 6 days of insulin treatment [62]. Substance P content of the adrenal medulla (as measured by RIA) also increased nearly seven-fold within 24 h in response to insulin-induced hypoglycaemia in rats [62]. However, another study using an in vivo dialysis system to measure the release rate of catecholamines and substance P from the adrenal gland in rats in response to various stressors had shown that insulin-induced hypoglycaemia did not alter the release rate of substance P from the adrenal gland [147]. Therefore, it is possible that the substance P expressed in response to insulin-induced hypoglycaemia is indeed not released from the adrenal gland. In rats, insulin-induced hypoglycaemia also significantly increased preproENK mRNA levels in the adrenal gland/medulla within hours [144,145,146]. Furthermore, PACAP increased TH activity within 4 h in insulin-treated mice, thereby aiding adrenaline secretion, an effect that was absent in the PACAP knockout mice which also showed impairments in TH induction and catecholamine synthesis [148]. Administration of insulin caused a dose-dependent lethality as early as 2 h from the time of insulin administration in the PACAP knockout mice, highlighting the crucial role of PACAP in restoring blood glucose levels during hypoglycaemia in mice [148].

### 4.3. Effects of Peptide Antagonists

In response to both single and recurrent hypoglycaemia, NPY immunoreactivity within the adrenal medulla increased significantly and inhibition of NPY signalling either transgenically (in NPY knockout mice) or pharmacologically using BIBP3226, an NPY antagonist, prevented the reduction in TH expression in the adrenal medulla in response to recurrent hypoglycaemia in mice [34]. The above results indicate that NPY could have a significant role in altering catecholamine secretion in response to insulin-induced hypoglycaemia, and thus in HAAF.

In response to insulin-induced hypoglycaemia, preproENK mRNA levels increased significantly in the adrenal gland/medulla rats [144,145,146] and opioids such as enkephalins and ꞵ-endorphins inhibited the catecholamine secretion from bovine adrenal chromaffin cells and rat adrenal glands [130,149,150]. Administration of naloxone during antecedent hypoglycaemia in non-diabetic and diabetic humans has successfully restored adrenaline response to subsequent hypoglycaemia [151,152]. Therefore, it is possible that naloxone blocked the inhibition of opioids at the level of adrenals or at the level of both adrenals and the central nervous system to restore the adrenaline response to subsequent hypoglycaemia in humans. In rat adrenal glands, the inhibitory effect of enkephalin on catecholamine release was prevented by naloxone [130]. In our laboratory, naloxone administration increased Ser31TH and Ser40TH phosphorylation in the adrenal gland in rats exposed to recurrent hypoglycaemia, indicating that opioids did have an inhibitory effect on TH phosphorylation in the adrenal gland in rats. Nevertheless, unlike human studies, naloxone administration did not restore plasma adrenaline response to subsequent hypoglycaemia in our study, or other rodent studies alike [97]. This was possibly because the opioid inhibition of catecholamine secretion from the adrenal was not mediated through the opioid receptors or due to the non-specific inhibitory effects of naloxone on nicotinic receptors [98].

The studies listed above indicate variations in the expression of multiple peptides within the adrenal gland in response to insulin-induced hypoglycaemia and most of them can potentially alter the catecholamine secretion from the adrenal medulla. However, there are not many studies to our knowledge that have investigated the potential role of these neuropeptides in reducing adrenaline secretion in response in HAAF.

## 5. Challenges in Investigating the HAAF phenomenon

The major obstacle in investigating the responses in diabetic rodents is the lack of a reliable model of HAAF that can reproduce both components of the counterregulatory failure—the blunted adrenaline and glucagon responses to recurrent hypoglycaemia. This may be partly due to the fact that diabetic rats treated with insulin to control their blood glucose during experimentation may undergo multiple episodes of undocumented antecedent hypoglycaemia before the HAAF protocol begins. This problem may potentially be lessened using the continuous glucose monitoring system in rodents; however, this system is quite expensive and not many laboratories can acquire this system due to the high costs.

Furthermore, some studies have reported that diabetic rats required very high insulin concentrations (30–40 IU/kg) to induce hypoglycaemia (via i.p. injections) [87]. Such high doses of insulin can make interpretations of such studies difficult.

To our knowledge, currently no studies have attempted to experimentally model single or recurrent hypoglycaemia in type 2 diabetes to assess its effects on mechanisms of the adrenal gland related to the release of adrenaline. This is important to model in future studies given the different mechanisms involved in the onset of hypoglycaemia-induced unawareness in type 1 versus type 2 diabetes.

The lack of HAAF model in mice has also delayed the progress in the field. Recently, an HAAF model in non-diabetic mice has been developed and validated [89]. This study opens up new exciting opportunities to investigate the HAAF mechanisms using genetically modified mice, although the results from this study should be confirmed by other laboratories. Progress in the field is further hindered by the sparsity of studies of the HAAF phenomenon in humans. A recent review has assessed experimentally induced HAAF in humans and outlined the important factors to consider when designing HAAF experiments, and thus may facilitate future studies in humans [19]. Rodent models, as well as clinical studies, of insulinomas are also encouraged as these would provide valuable insight into the complex mechanisms involved in HAAF. It would also be advantageous to develop a diabetic HAAF model in mice to move the field forward. Finally, given the lack of a reliable model of HAAF in rodents, in vitro studies may further elucidate the complexity of the HAAF phenomenon, although this presents a challenge given the multiple organ systems involved. Unfortunately, it is impossible to mimic HAAF in cell lines, because glucose sensing occurs in the brain [153] and peripheral sites [154], but no glucose sensing has been described in the chromaffin cells yet. However, some studies have isolated chromaffin cells from hypoglycaemic mice and have shown that the consequences of recurrent hypoglycaemia are detectable in isolated chromaffin cells in vitro [34].

## 6. Summary and Conclusions

HAAF in diabetic patients occurs as a result of more frequent and severe hypoglycaemic episodes due to impairments in the adrenomedullary response and neurogenic symptom response. This can occur from a resultant overdose of exogenous insulin since loss of pancreatic β-cells (type 1 diabetes) or pancreatic insulin insufficiency and insulin resistance (late-stage type 2 diabetes) do not sufficiently correct hypoglycaemic states following the administration of exogenous insulin. Such recurrent episodes of hypoglycaemia lead to hypoglycaemic unawareness and impairments in the adrenomedullary response. In this review, we mainly focused on the adrenal medulla biology, function, and the responses to single and recurrent hypoglycaemia in diabetic and non-diabetic subjects.

The main points are summarised below

1. The effects of recurrent hypoglycaemia or diabetes on the adrenomedullary signalling, synaptic proteins, and catecholamine release are not established, and the studies are very limited in this area.
2. The catecholamine synthetic capacity of the adrenal medulla in response to recurrent hypoglycaemia has been extensively studied in non-diabetic animals, but produced inconsistent results. Short-term changes in relation to TH phosphorylation, specifically at Ser31 and Ser40 has not been consistently shown. Findings of long-term changes in relation to TH protein expression show that rats have increased TH protein expression following single or recurrent hypoglycaemia, while studies in mice show that TH protein levels return to basal levels in the context of recurrent hypoglycaemia, indicating that the catecholamine synthetic response of the adrenal medulla to recurrent hypoglycaemia may be species specific or experimental design specific. Studies in diabetic rodents are very limited.
3. While there are very few studies which have investigated the effects of insulin-induced hypoglycaemia on neuropeptides in the adrenal gland, current evidence suggests that recurrent insulin-induced hypoglycaemia increases protein expression of neuropeptides such as NPY, galanin, and proenkephalin. Moreover, this phenomenon may occur across different species since consistent findings have been reported in both rats and mice. Peptide antagonism, particularly for NPY and opioids, could be a key strategy in preventing the short-term and long-term changes to TH expression and activity. Despite some variations in the reported expression of the neuropeptides covered in this review, their collective impact on adrenaline secretion in HAAF warrants further investigation.
4. Animal models of HAAF in diabetic rodents are still lacking.

We would like to emphasise that investigating the effects of single and recurrent hypoglycaemia in non-diabetic rodents has been extremely important and significantly improved our knowledge about the HAAF phenomenon. However, ultimately, in order to improve the lives of people with diabetes, we need to investigate the HAAF mechanisms in diabetic rodents. However, these studies have been very limited and mainly performed in rats.

## 7. Future Directions

There are still some gaps in the field regarding the role of adrenal medulla in HAAF and the following questions need to be answered.

1. How catecholamine synthesis and release from the adrenal medulla are altered in response to recurrent hypoglycaemia in diabetic and non-diabetic subjects?
2. Do adrenomedullary peptides contribute to HAAF?
3. What are the cellular and molecular mechanisms that are involved in adrenal medullary responses to single and recurrent hypoglycaemia in diabetic and non-diabetic subjects?

It is hoped that further refinements in the diabetic rat model of HAAF and development of the diabetic mice model of HAAF will bring new insights into the HAAF phenomenon that can facilitate the identification of novel targets and development of new treatments for patients with type 1 diabetes who develop HAAF.

## Figures and Tables

**Table 2:** Evidence of co-existence of different neuropeptides in the adrenal chromaffin cells and their possible effects on catecholamine secretion in different species (when administered exogenously). PACAP, pituitary adenylate cyclase activating peptide; VIP, vasoactive intestinal peptide; NPY, neuropeptide Y; ENK, enkephalin; SP, substance P; CGRP, calcitonin gene-related peptide.

Peptide	Species	Model System	Immunoreactivity in Adrenaline Chromaffin Cells	Immunoreactivity in Noradrenaline Chromaffin Cells	Effects on Catecholamine Release
PACAP	Rat [115,116,117] Mouse [116] Human [118] Bovine [119] Porcine [120,121]	Adrenal gland [115,116,117,118] Adrenal medullary chromaffin cells [119,120,121]	+ [118] − [115,116]	+ [115,116,118]	↑ [117,118,119,120,121]
VIP	Rat [122] Human [118]	Adrenal gland [118,122]	+ [118]	+ [118]	↑ [118,122]
NPY	Rat [116,123] Human [124] Bovine [125,126]	Adrenal gland [116,123] Adrenal medullary chromaffin cells [124,125,126]	+ [116,123,126]	+ [123] − [116,126]	↑ [124] ↓ [125]
Neurotensin	Bovine [127] Cat [128]	Adrenal medullary chromaffin cells [127] Adrenal medulla [128]	− [128]	+ [128]	↑ [127]
Galanin	Rat [129]	Adrenal gland [129]	+ [129]	+ [129]	↑ [129]
ENK	Rat [130,131] Bovine [132]	Adrenal gland [130,131] Adrenal medulla [132]	+ [131,132]	− [131,132]	↓ [130]
SP	Guinea pig [133] Rat [134]	Adrenal medullary chromaffin cells [133] Adrenal gland [134]	+ [133]	+ [133]	↓ [133,134]
CGRP	Rat [135]	Adrenal gland [135]	+ [135]	− [135]	↓ [136]
Somatostatin	Guinea pig [133]	Adrenal medullary chromaffin cells [133]	+ [133]	+ [133]	↓ [133]

+, indicates positive immunoreactivity; −, indicates absent/reduced immunoreactivity; ↓ indicates a decrease; ↑ indicates an increase.

**Table 3:** Effects of insulin treatment on the gene expression and content of the peptides in the adrenal gland/medulla in rodents. Ir, immunoreactivity; NPY, neuropeptide Y; PreproENK, preproenkephalin; CGRP, calcitonin gene-related peptide; SP, substance P.

Peptide	Species	Stimulus	Effects on Gene/Peptide Expression in the Adrenal Gland/Medulla
NPY	Rats [37,142,143]	Recurrent hypoglycaemia (2 U/kg twice daily for three days) [37]	↑ ProNPY gene [37]
		Insulin (2 U/kg) [142]	↑ mRNA and peptide [142]
		Insulin (0.7, 3.5, 7 U/kg) [143]	Dose-dependent increase in mRNA; ↑ NPY-ir after 7 U/kg insulin [143]
	Mice [34]	Recurrent hypoglycaemia (2.5 U/kg once daily for 3 consecutive days) [34]	↑ NPY-ir [34]
Enkephalin	Rats [37,144,145,146]	Recurrent hypoglycaemia (2 U/kg twice daily for three days) [37]	↑ Proenkephalin gene [37]
		Insulin (10 U/kg) [144]	↑ PreproENK mRNA [144]
		Insulin (8.5 U/kg) [145]	↑ PreproENK mRNA [145]
		Insulin (10 U/kg) [146]	↑ PreproENK mRNA [146]
CGRP	Rats [142]	Insulin (2 U/kg) [142]	↑ CGRP mRNA and Peptide [142]
SP	Rats [62]	Insulin (5 U/kg) [62]	↑ SP peptide [62]
Galanin	Rats [37,62,146]	Recurrent hypoglycaemia (2 U/kg twice daily for three days) [37]	↑ Galanin prepropeptide gene [37]
		Insulin (5 U/kg) [62]	↑ Galanin peptide [62]
		Insulin (10 U/kg) [146]	↑ Galanin mRNA [146]
Neurotensin	Rats [37,62]	Recurrent hypoglycaemia (2 U/kg twice daily for three days) [37]	↑ Neurotensin gene [37]
		Insulin (5 U/kg) [62]	↑ Neurotensin peptide [62]

↑ indicates an increase.

## Data Availability

Not applicable.
